# Immortalization of human primary prostate epithelial cells via CRISPR inactivation of the *CDKN2A* locus and expression of telomerase

**DOI:** 10.1038/s41391-020-00274-4

**Published:** 2020-09-01

**Authors:** Ziran Zhao, Holly Fowle, Henkel Valentine, Zemin Liu, Yinfei Tan, Jianming Pei, Simone Badal, Joseph R. Testa, Xavier Graña

**Affiliations:** 1 Fels Institute for Cancer Research and Molecular Biology, Philadelphia, PA USA; 2 Department of Basic Medical Sciences, Faculty of Medical Sciences Teaching and Research Complex, The University of the West Indies, Mona, Jamaica; 3 Fox Chase Cancer Center, Temple Health, Philadelphia, PA USA

**Keywords:** Prostate cancer, Cancer genetics

## Abstract

**Background:**

Immortalization of primary prostate epithelial cells (PrEC) with just hTERT expression is particularly inefficient in the absence of DNA tumor viral proteins or p16^INK4A^ knockdown.

**Materials and methods:**

Here, we describe the establishment of immortalized normal prostate epithelial cell line models using CRISPR technology to inactivate the *CDKN2A* locus concomitantly with ectopic expression of an h*TERT* transgene.

**Results:**

Using this approach, we have obtained immortal cell clones that exhibit fundamental characteristics of normal cells, including diploid genomes, near normal karyotypes, normal p53 and pRB cell responses, the ability to form non-invasive spheroids, and a non-transformed phenotype. Based on marker expression, these clones are of basal cell origin.

**Conclusions:**

Use of this approach resulted in the immortalization of independent clones of PrEC that retained normal characteristics, were stable, and non-transformed. Thus, this approach could be used for the immortalization of normal primary prostate cells. This technique could also be useful for establishing cell lines from prostate tumor tissues of different tumor grades and/or from patients of diverse ethnicities to generate cell line models that facilitate the study of the molecular basis of disease disparity.

## Introduction

Prostate cancer is the most common cancer diagnosed in men, accounting for an estimated 20% of new cancer cases and 10% of cancer deaths in US males in 2020 [1]. Prostate cancer also represents one of the largest cancer disparities of mortality rates between non-Hispanic blacks and whites [2]. However, the molecular basis of prostate cancer development and these disparities remains unclear, at least in part due to the limitations of available cellular models. Unlike other cancer types, it has been particularly challenging to develop prostate cell line models in culture [3]. The most widely used cell lines, PC-3, DU145, and LNCaP, were established spontaneously and all derived from metastases [4]. Moreover, they have already acquired numerous genomic alterations [5]. To help define the minimal relevant genetic alterations needed for prostate cell transformation and 3D organoid aberrant growth, it is necessary to generate a collection of immortal normal prostate cell lines and develop a methodology that can be easily used for the efficient establishment of primary prostate cancer cells that often fail to establish in culture.

Prostate epithelial cells (PrEC) can only go through a limited number of passages before they become senescent, which is defined as a process in which cells stop dividing and undergo distinctive phenotypic changes [6]. Immortalization allows cells to evade senescence and continue to divide. Primary PrEC have been previously immortalized using a human telomerase reverse transcriptase (h*TERT*) transgene with limited success [7–9]. Most of these cell lines are unavailable, as they have been lost or do not show their described original characteristics. One of these cell lines, EP156T, has apparent normal epithelial features [8], but exhibits karyotypic abnormalities of unknown effects [10], which is consistent with further alterations being needed for immortalization. Of note, in two studies that generated hTERT-immortalized PrEC with near diploid karyotypes, the resulting cells lost expression of *CDKN2A/*p16^INK4A 8,9^. In one case, this was correlated with increased methylation of the p16^INK4A^ promoter [9]. In accordance with a potential requirement for p16^INK4A^ loss, Hahn and collaborators have shown that PrEC can be immortalized with ectopic expression of hTERT with co-expression of large T antigen, which inactivates the tumor suppressors pRB and p53 [11]. pRB is downstream of p16^INK4A^, and both pRB and p53 are needed to establish a permanent cell cycle arrest in response to senescence signals [12, 13]. Others have shown that p16^INK4A^ expression increases with PrEC passage [14–16]. Consistent with these observations, shRNA mediated knockdown of p16^INK4A^ cooperates with hTERT expression in the immortalization on PrEC [16]. More recently, it has been shown that hPrEC at very early passage (<7) can be immortalized via expression of hTERT alone when p16^INK4A^ expression is relatively low [14], but this limits the window of opportunity for immortalization of primary cells. Moreover, inactivation of p53 function, presumably downstream of p14^ARF^, with a dominant negative mutant generates immortal cells that grow more efficiently [16]. Therefore, elimination rather than attenuation of p16^INK4A^/p14^ARF^ expression may increase the efficiency of immortalization and at the same time eliminate pressure for selection of genetic alterations that could attenuate senescence as well as passenger alterations that are not required for immortalization but contribute to the selection of karyotypically deficient cells.

Thus, we devised a strategy to rapidly attenuate senescence signals simultaneously with the expression of hTERT to prevent spontaneous alterations that are dispensable for immortalization via CRISPR-mediated inactivation of the *CDKN2A* locus, which directs the expression of both p16^INK4A^ and p14^ARF^ [12, 17]. This method facilitates direct immortalization of PrEC without depending on the spontaneous silencing of p16^INK4A^ expression or p16^INK4A^/p14^ARF^ shRNAs, which may not be able to stably maintain low expression under all growth conditions. The immortal cell clones described here are of basal prostate cell origin and retain the characteristics of normal cells including normal p53 and pRB pathways, and near normal karyotypes.

## Materials and methods

### Cell culture

HPrEC (ATCC PCS-440-010), immortalized hPrEC *T-ΔN2A* clones, and EP156T cells (ATCC CRL-3289) were cultured in prostate epithelial medium supplemented with growth factors (ATCC PCS-440-040). All other cell lines were obtained from ATCC and tested for mycoplasma regularly.

For p53 pathway analysis, cells were treated with 150 nM flavopiridol, 60 µM etoposide, or 1 µg/mL doxorubicin and collected at the indicated time points. For contact inhibition assays, 150,000 cells were seeded in 6 cm plates, collected at indicated days and analyzed for western blotting and cell cycle DNA-content by PI/flow cytometry as described earlier [18].

For 3D spheroid culture, procedures were adapted from [19]. For hPrEC spheroids, Growth Factor Reduced Matrigel Matrix (Corning, REF 356231) was supplemented as described previously [20]. The bottom layer of Matrigel was 75% while the top was 50%. 2,000 cells of EP156T, hPrEC T-ΔN2A clone 1 or clone 2 were seeded in each well inbetween the Matrigel layers. Supplemented medium was added on top and replaced every other day for about two weeks.

Anchorage independent assays and clonogenic assays were performed as described earlier [21]. The bottom layer contained 0.6% agar. For clonogenic assays, 170 cells were seeded into a 6-well plate.

### Plasmids and viral transduction and DNA analysis

LentiCRISPRv2-sg*CDKN2A* vector was generated as described [22] using oligos: sg*CDKN2A*_FWD: CACCGTGCACGGGTCGGGTGAGAG and sg*CDKN2A*_REV: AAAC CTCTCACCCGACCCGTGCAC.

To immortalize hPrEC, cells were cotransduced with lentiviral pLV-hTERT-IRES-hygro (Addgene#85140) and lentiCRISPRv2-sg*CDKN2A* targeting exon 2 of *CDKN2A* (GTGCACGGGTCGGGTGAGAG) and selected with 25 µg/mL hygromycin and 0.25 µg/mL puromycin.

Telomere length was quantified by qPCR using the Absolute Human Telomere Length Quantification qPCR Assay Kit (ScienCell, Cat #8918) as per manufacturer instructions.

To verify *CDKN2A* targeting in immortalized clones, PCR was performed using primers targeting *CDKN2A* exon 2 (*CDKN2A* FWD: CTG TGC TGG AAA ATG AAT GC; *CDKN2A* REV: CTG GAA GCA AAT GTA GGG G) with annealing temperature at 55 °C. Globin primers were used as control (Globin FWD: CAA CTT CAT CCA CGT TCA CC; Globin REV: GAA GAG CCA AGG ACA GGT AC).

### Immunoblots, immunofluorescence imaging and confocal microscopy

Western blot analysis was performed as previously described [23], using antibodies indicated in Supplementary Tables 1 and 2. Cells for 3D spheroid culture were seeded in iBidi µ-slides (ibidi #81501). For immunofluorescence detection, spheroids were washed with PBS and fixed with 30 µl fixation/permeabilization solution consisting of 2% paraformaldehyde, 0.3% Triton X-100, 5 mM EGTA, and 1 mM MgCl_2_ in PBS at room temperature for 20 min. Spheroids were washed with PBS three times, followed by blocking with 20% horse serum in PBS with 0.05% Tween 20 (PBS-T) at room temperature for 60 min. Samples were incubated with primary antibodies in PBS-T for 60 min at room temperature or at 4 °C overnight, washed with PBS three times, followed by fluorescence-conjugated secondary antibodies mixed with phalloidin for 60 min at room temperature. After rinsing three times with PBS, DAPI was applied to samples for 15 min. Samples were protected from light until imaging with a Leica TCS SP8 confocal microscope with 63x lens.

### Cytogenetic analysis and chromosome microarray analysis (CMA)

To determine the karyotype of the immortalized clones, exponentially growing cells were treated with 0.01 µg/ml colcemid as previously described [24]. Chromosome spreads were prepared and G-banded according to standard procedures [25]. For each clone, at least 10 metaphases were examined. CMA was performed using Thermofisher microarray CytoScan™ HD array (https://www.thermofisher.com/us/en/home/life-science/microarray-analysis/cytogenetics-analysis-microarrays.html), which contains more than 2.6 million copy number markers of which 750,000 are “genotype–able” SNPs and 1.9 million are nonpolymorphic probes with 250 ng of total genomic DNA from each test sample as described earlier [26].

## Results

### Generating immortalized human prostate epithelial cell line

**Fig. 1:**
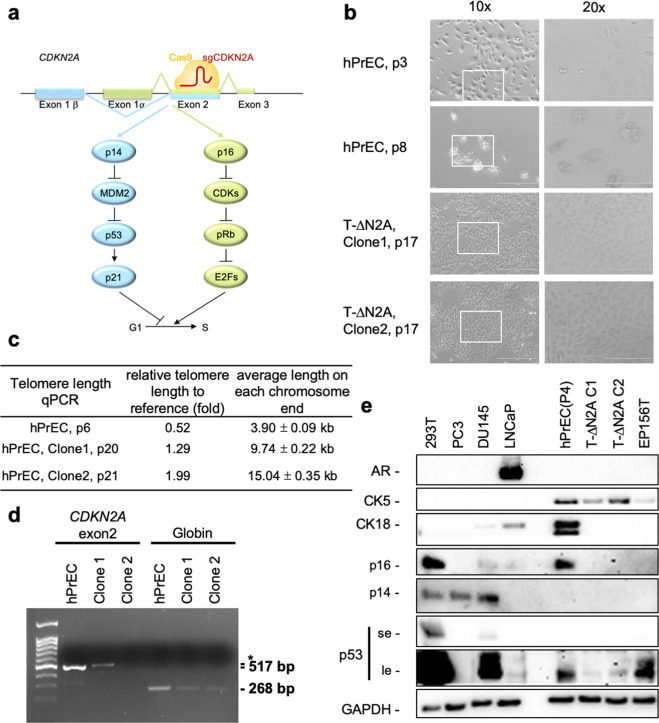
Immortalization and verification of human prostate epithelial cells (PrEC). **a** Scheme of the *CDKN2A* gene locus, which encodes p14^ARF^ and p16^INK4A^, and the targeted senescence pathway. The sgRNA targets exon 2, which encodes a segment of both proteins using alternative frames. **b** Morphology of primary hPrEC in culture and clones obtained upon selection with antibiotics. Passage numbers are indicated. **c** Telomere length qPCR with relative and absolute length of telomeres in primary hPrEC and immortalized clones. **d** PCR to amplify *CDKN2A* exon 2, which was targeted by CRISPR sgRNA. **e** Western blot of p16^INK4A^, p14^ARF^ and additional cell markers including those for basal (CK5) and luminal (CK18) tissue origin.

Our attempts to establish immortalized primary normal human PrEC via ectopic expression of hTERT alone failed to generate any immortal clones following selection. This indicated that the introduction of a single h*TERT* transgene is very inefficient as a single hit for immortalization of PrEC, supporting why long-term successful stable immortalization of PrEC has been rarely accomplished [8]. Thus, we rationalized that ablation of the *CDKN2A* locus (Fig. 1a), which encode p16^INK4A^ and p14^ARF^, two gene products known to be activated in response to senescence signals that drive pRB and p53 activation, would prevent senescence and facilitate rapid immortalization precluding spontaneous alterations. We also wanted to promote immortalization without using viral oncogenes that may have additional targets [27]. Moreover, we also rationalized that by not directly altering the pRB and p53 tumor suppressors, other properties of normal cells including cell cycle arrest in response to growth to high density, DNA damage, and other cellular stresses would be preserved.

To ablate *CDKN2A* in hPrEC, we designed a guide RNA (sg*CDKN2A*) for Cas9 to target exon 2 of *CDKN2A*, which contributes sequences for both p16^INK4A^ and p14^ARF^ (Fig. 1a). hPrEC were transduced with lentiviruses expressing h*TERT* and/or sg*CDKN2A* and selected with hygromycin and/or puromycin. One *CDKN2A* knockout clone (ΔN2A) and two clones of the combination of ectopic hTERT expression and *CDKN2A* knockout (T-ΔN2A) were successfully selected. No clones survived selection after transduction with hTERT lentiviruses alone upon multiple attempts. Non-transduced primary hPrEC grew sparsely with cells varying in sizes at passage 3 (Fig. 1b). Starting from passage 7, the majority of hPrEC showed senescence characteristics with enlarged cell nuclei and flat morphology. At passage 8, few cells remained attached. The ΔN2A clone started to show signs of senescence at passage 8 and became fully senescent at passage 9, leaving an insufficient number of cells for further characterization experiments. In contrast, two T-ΔN2A clones proliferated relatively fast, showing compact and smaller cells (Fig. 1b). The derived cell lines have been continuously grown and are currently at passage 36 without showing any signs of senescence.

### Characterization of the immortalized cell lines

We next verified that the immortalized cell clones had the intended *CDKN2A* exon 2 deletion and expressed the hTERT transgene. qPCR performed with primers targeting the telomere and a single copy reference using genomic DNA showed that the relative telomere length in the immortalized clones ranged from 1.29- to 1.99-fold greater than the reference human genomic DNA, while the corresponding hPrEC ratio was only 0.52-fold (Fig. 1c). This strongly suggests that ectopic hTERT expression elongates/maintains telomeres in immortalized hPrEC *T-ΔN2A* clones. Next, we used PCR to amplify the region of *CDKN2A* exon 2 flanking the guide RNA targeting site cut by Cas9. Comparing with the band amplified from hPrEC genomic DNA, clone 1 produced a *CDKN2A* PCR product migrating slightly slower than the control PCR product, indicating the presence of a small insertion into the Cas9 lesion during DNA repair (Fig. 1d). There were no amplified PCR products from clone 2 genomic DNA, suggesting that the genomic region deleted is larger than the region selected for amplification (Fig. 1d). As expected, primers for the human globin gene amplified a band of the expected size for all samples. Therefore, the *CDKN2A* gene was efficiently targeted by Cas9 and edited in both clones.

Next, we wanted to confirm that no products were expressed from the targeted *CDKN2A* locus. HEK293T cells were used as a positive control for p16^INK4A^ and p14^ARF^ expression, and a panel of PCa cell lines and EP156T, the only available hTERT-immortalized prostate epithelial cell line [8], as controls for other cell markers. We detected p16^INK4A^ expression in hPrEC at passage 4, but no expression of p16^INK4A^ was detected in either immortalized clone (Fig. 1e), suggesting that the CRISPR knockout efficiently prevented p16^INK4A^ expression. p14^ARF^ and p16^INK4A^ expression were absent in the two ΔN2A clones. p14^ARF^ expression was also not detected in hPrEC at passage 4, which may not be activated at early cell passages (note that these cells show p53 activation likely via a p14^ARF^-independent mechanism). However, p14^ARF^ was readily detectable in PC3 and DU145 PCa cell lysates. These results confirm that CRISPR deletions targeting exon 2 efficiently eliminated expression of the two *CDKN2A* protein products.

hPrEC and its derived T-ΔN2A clones expressed high levels of basal cell markers cytokeratin 5 and p63 (Fig. 1e and Supplementary Fig. 1A) and undetectable levels of luminal cell marker cytokeratin 18 and AR (Fig. 1e and Supplementary Fig. 1B). AR was not detected even after growth in the presence of 2 nM DHT and upon 24 h following EGF withdrawal [20]. This indicates that hPrEC-T-ΔN2A clones, like EP156T cells, are derived from prostate basal epithelial cells.

The karyotype of EP156T cells was reported to be 46–48,XY, +2[2], −8[3], +13[4], −20[2], +der(20)[10], +mar[3][cp10] [10]. The “cp” designation refers to composite karyotype, which is indicative of significant to great karyotypic heterogeneity among the cells examined, although different cells shared some cytogenetic features. The composite karyotype contains all clonally-occurring abnormalities, with the total number of cells in which each clonal change was observed given in separate brackets. In EP156T, all 10 karyotyped metaphases had a derivative chromosome 20, der(20), that apparently contained translocated chromosomal material of uncertain origin.

**Fig. 2:**
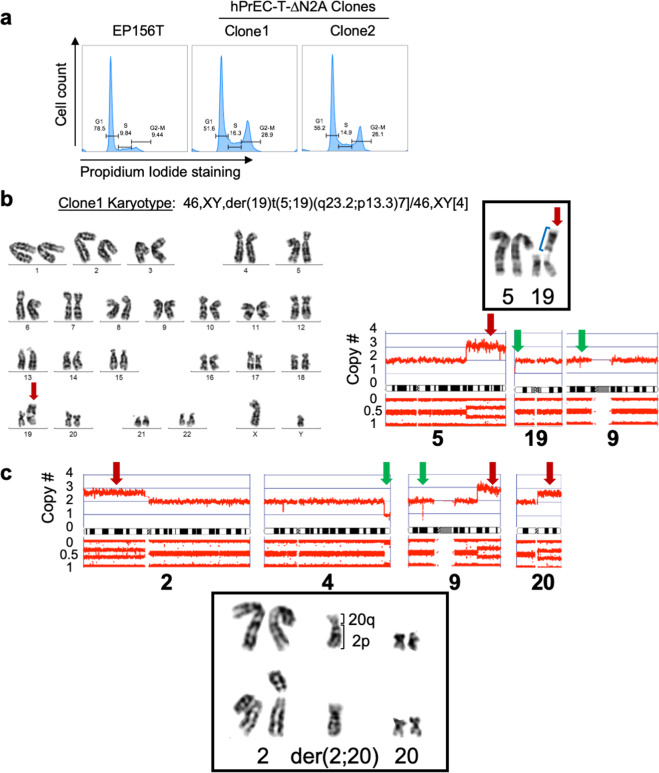
Characterization of immortalized human prostate epithelial cell line. **a** PI staining and cell cycle analysis. **b** Karyotypes of hPrEC-*T-ΔN2A* clone 1. Full karyotypes of Clone 1 (*left*) and partial karyotype of chromosomes 5 and 19 from another metaphase with more elongated chromosomes (inset, *right*) are shown. CMA profiles of chromosomes 5, 19, and 9 are shown below the inset. **c** Clone 2 CMA profiles of chromosomes 2, 4, 9, and 20 (top) and partial karyotype of chromosomes 2 and 20 (inset, *bottom*). Arrows indicate abnormalities, with red arrows indicating gains and green arrows indicating deletions.

**Fig. 3:**
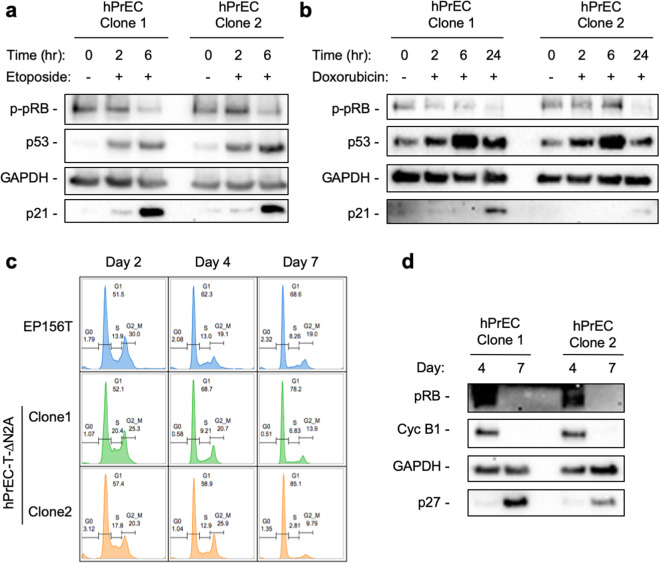
The immortalized human prostate epithelial cell lines have intact p53 pathway and normal contact inhibition. **a**, **b** Treatment with cytotoxic agents etoposide (60 µM) and doxorubicin (1 µg/mL) increased p53 and p21 expression in hPrEC-*T-ΔN2A* clones as determined by western blot analysis. **c** Growth to high cell density results in cell cycle exit. EP156T cell line and hPrEC-*T-ΔN2A* clones were allowed to growth to confluency. Cells were collected at the indicated times (in days) and cell cycle arrest was detected by measuring DNA content by PI/flow cytometry analysis (**c**). G0 (quiescence) and mitotic markers were determined by western blot analysis (**d**). Experiments shown are representative of three independent experiments unless indicated.

To determine if the immortalized *T-ΔN2A* clones maintained the features of normal cells, we examined their cell cycle parameters and karyotype. Cells from exponentially growing *T-ΔN2A* clones were stained with propidium iodide and analyzed by FACS. Similarly to immortalized EP156T cells, both T-ΔN2A clones lacked significant polyploidy, suggesting that the cells were diploid (Fig. 2a). Cytogenetic analysis showed that the karyotype of Clone 1 is mosaic: 46,XY,der(19)t(5;19)(q23.2;p13.3)[7]/46,XY[4], including cells with normal karyotype and cells with an abnormal chromosome 19, der(19), that involves an unbalanced translocation with the distal end of the long arm of chromosome 5, 5q23.2→qter. A representative karyotype of the cells in Clone 1 is shown in Fig. 3b, which also includes an inset showing the der(19) from another metaphase with more elongated chromosomes. Chromosome microarray analysis (CMA) confirmed that the der(19) was an unbalanced rearrangement with a gain of 5q23.2→qter, as there were three copies of nucleotides chr5:121,598,528–180,719,789 and loss of one copy of a small segment in chromosome band 19p13.3, which includes nucleotides chr19:260,911–683,931 (Fig. 2b and Supplementary Fig. 2). Notably, the CMA profile also revealed a focal deletion in 9p21.3, (9p21.3)x1, which appeared to result in loss of one copy of the *CDKN2A* locus in about 50% of the cells (Fig. 2b and Supplementary Fig. 2). Cytogenetic analysis of Clone 2 was limited by the low number of mitotic cells but revealed a mosaic karyotype in two separate metaphase harvests: 47,XY, +der(2;20)(p10;q10), +0–1mar[9]/46,XY[1]. This is consistent to what was observed by CMA analysis, which showed gains of 2p and 20q, i.e., (2p)x3 and (20q)x3 (Fig. 2c). The CMA analysis also revealed a gain of 9q31.1q34.3, which might be present in an unidentifiable marker chromosome noted in some metaphases on both occasions. Another abnormality seen in the CMA analysis, (4q34.3q35.2)x1, could not be identified with certainty in the available metaphases. Interestingly, the CMA profile also revealed a focal deletion in 9p, (9p21.3)x0, which resulted in homozygous loss of the *CDKN2A* locus. The abnormalities of 4q and 9p are shown in Fig. 2c and Supplementary Fig. 3.

Since clone 1 had a significant proportion of karyotypically normal cells, a chromosome 5q32 *PDGFRB* break apart FISH probe (MetaSystems, Newton, MA) was then used to screen subclones of Clone 1 that were generated by single cell dilution. However, all subclones exhibited 3 fused orange-green signals, indicating three copies of the 5q32 region (Supplementary Fig. 4), with no subclones being present without this alteration. Therefore, it seems that the cells with the der(19) had outgrown the normal diploid cells by the time the cells were diluted to generate single cell clones. Note that single cell cloning started at passage 34 and involved >20 additional population doublings with no signs of senescence observed.

To determine if the immortalized hPrEC clones retain the properties of normal cells, we determined their response to signals that activate the p53 and/or the pRB pathway. First, we treated the cells with flavopiridol to induce p53 expression [28]. The expression of p53 dramatically increased 2 h after flavopiridol treatment, returning to near basal levels by 24 h (Supplementary Fig. 5), suggesting that p14^ARF^-independent p53 signaling is not affected. We also assessed if the p53 pathway was intact in T-ΔN2A clones upon treatment with DNA-damaging agents. As shown in Fig. 3a, treatment of T-ΔN2A clones 1 and 2 with etoposide resulted in p53 upregulation by 2 h and subsequent potent upregulation of p21 by 6 h. Doxorubicin treatment resulted in potent upregulation of p53 expression that peaked at 6 h, followed by p21 upregulation at 24 h (Fig. 3b). This demonstrates that T-ΔN2A clones retain normal p53/p21 signaling in response to DNA damage.

Next, we determined whether growth to high cell density resulted in G1/G0 cell cycle arrest, which is known to trigger activation of pRB and related proteins [29, 30]. Accumulation of cells with a G0/G1 DNA content was already observed with increased cell density at day 4 prior to reaching cell confluence, and prominent G0/G1 arrest was observed by day 7 when cells were fully confluent (Fig. 3c). Consistent with these results, the levels of mitotic cyclin B1 were undetectable and total pRB expression was downregulated while p27 expression was upregulated at day 7 compared to day 4 in both *T-ΔN2A* clones (Fig. 3d). We have consistently observed that these cells are highly sensitive to cell-to-cell contact inhibition of proliferation and when seeded at low concentration, they form colonies that become quiescent even if these colonies do not fully cover the plate surface (data not shown). Thus, our results indicate that the cells are strongly arrested by cell contacts, which is consistent with their epithelial origin.

**Fig. 4:**
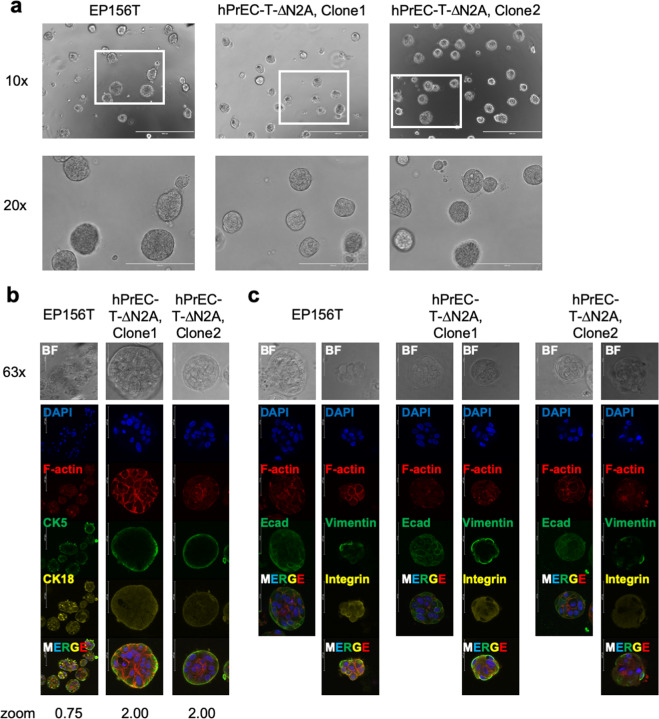
Characterization of immortalized human prostate epithelial cell lines in 3D culture. **a** Images of EP156T and hPrEC-T-ΔN2A spheroids were taken at Day 13 using EVOS inverted microscope. Spheroids were IF stained with (**b**) keratin and (**c**) EMT markers and imaged with confocal microscopy, 63x. Zoom factor of each image was as indicated. Experiments shown are representative of two independent experiments unless indicated.

To explore the differentiation potential of these immortalized cells and their ability to form normally shaped spheroids, we seeded them in Matrigel. T-ΔN2A clones and EP156T cells were cultured according to methodology based on previously described work [19, 20]. EP156T and hPrEC-T-ΔN2A clones were able to establish round and similarly sized spheroids in Matrigel (Fig. 4a). After 13 days in culture, spheroids were fixed and immunofluorescence stained for prostate epithelial cell markers. The spheroids consisted of multiple cells, and individual cells were enclosed with F-actin. The boundary layer of the spheroids was high in CK5 and low in CK18 (Fig. 4b), indicating that both hPrEC-T-ΔN2A clones were polarized and of basal cell origin (consistent with the results in Fig. 1e).

We also stained the spheroids for Epithelial-Mesenchymal Transition (EMT) markers. It was reported that EP156T exhibited high E-cadherin and low vimentin expression in 2D culture [10]. Under our spheroid culture conditions, we observed positive E-cadherin staining in EP156T and both *T-ΔN2A* clones. Of note, vimentin staining was positive in a subset of cells facing the ECM, exhibiting clear polarity in EP156T and both immortalized T-ΔN2A clones (Fig. 4c). We did not detect expression of AR, even after EGF withdrawal [20].

**Fig. 5:**
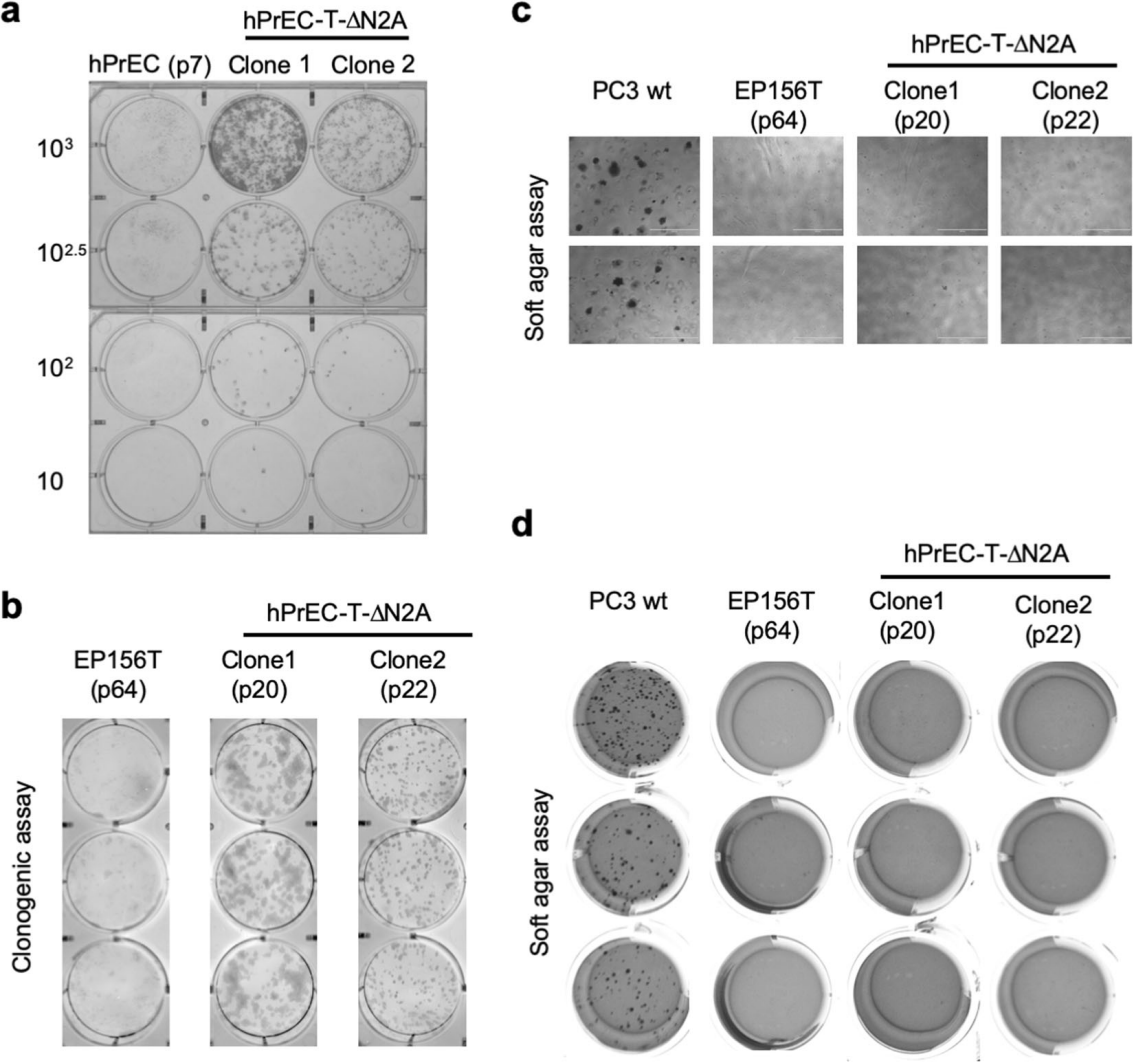
Clonogenic and transformative assays of immortalized hPrEC. Two independent clonogenic assays stained with crystal violet at Day 7 (**a**) and Day 9 (**b**) post seeding. **c**. Soft agar assay in culture at Day 16, 4x. D. Soft agar assay stained with crystal violet at Day 28. Experiments shown are representative of two independent experiments unless indicated.

Finally, clonogenic assays showed efficient colony formation by both T-ΔN2A clones, generating the expected number of colonies based on cell seeding. In contrast, hPrEC seeded at passage 7 failed to form visible colonies, consistent with these cells undergoing replicative senescence (Fig. 5a, b). Soft agar anchorage independence assays demonstrated that the cells are not transformed. Specifically, PC3 prostate cancer control cells formed numerous colonies as expected [21], while the immortalized clones remained mostly as single cells and were unable to form colonies (Fig. 5c, d).

## Discussion

This report describes a method for the efficient immortalization of normal prostate epithelial cells that could be used to build a collection of immortal normal PrEC from men with diverse genetic ancestry. We have used the same methodology with a biopsy of primary cells from a prostate tumor and successfully established a cell line (Badal and collaborators, in preparation). Therefore, this methodology could also be used to immortalize patient-derived prostate cancer cells from patients with diverse genetic ancestry, which is a major limitation of the existing PCa cell line collection. Such cell lines could potentially serve as cellular models to help address PCa disparities [31, 32].

Our preliminary data indicated that introduction of a single h*TERT* transgene is extremely inefficient as a single hit for immortalization of primary prostate epithelial cells from normal or cancer tissue. Of the three hTERT immortalized primary “normal” cell lines initially reported by others [7–9], one has a very abnormal karyotype [7], one is unavailable [9], and the one that is readily available shows the properties of normal cells [8] but exhibits some cytogenetic abnormalities and heterogenicity [10]. Two of these cell lines have lost expression of the tumor suppressor p16^INK4A^ during immortalization. Previous work has shown that senescent primary human fibroblasts and epithelial cells do not reenter the cell cycle upon hTERT expression. In contrast, senescent cells with low, but not high, expression of p16^INK4A^ reenter the cell cycle following inactivation of p53 [33]. Since primary PrEC transduced to express hTERT become senescent and fail to efficiently immortalize, and this can be bypassed by expression of SV40 large T antigen, which inactivates p53 and pRB [11], it appears that either hTERT cannot re-stabilize telomeres quickly enough to prevent senescence or that senescence is triggered by other environmental cues that activate p14^ARF^, the upstream activator of p53 [34]. Consistent with these results, it has been shown that shRNA-mediated knockdown of p16^INK4A^ allows hTERT immortalization of PrEC, and in this context, elimination of p53 function with a dominant-negative p53 mutant generates immortal PrEC that proliferate faster [16], suggesting that telomere independent senescent signaling through p53, possibly mediated by p14^ARF^ upregulation, can still attenuate the proliferation of these cells.

Based on these reports and our preliminary work, we hypothesized that expression of hTERT simultaneously with the co-inactivation of the *CDKN2A* locus would bypass the senescence signals that preclude immortalization in normal prostate epithelial cells. Our results support this hypothesis and provide a greatly improved methodology for immortalization that could also be applied to other cell types that prove difficult to immortalize. It remains to be determined if simultaneous inactivation of both p16^INK4A^ and p14^ARF^ is absolutely required, but based on previous observations this appears likely [11, 16, 33, 34]. Moreover, alterations of the *CDK2N2A* locus are detected at about 2% of PCa [35–38], suggesting that these alterations are also selected in prostate tumors in vivo. It is tempting to speculate that primary cells from tumors with alterations in the *CDKN2A* locus that already express hTERT may spontaneously establish in culture.

The cell lines described here provide a starting model for stepwise transformation assays aimed at determining prostate-relevant oncogene/tumor suppressor gene cooperativity as well as ECM invasion in 3D organoids. Moreover, the immortalization methodology could be applied to the rapid immortalization of cells in tumors and adjacent tumor tissue for studies of organoid formation and drug response. Finally, immortalization results in stable cell lines that retain the characteristics of the cells of origin, but are much easier to grow and cost effective for long term passage and further manipulation. This should facilitate establishment of new cell lines even in laboratories with limited resources around the globe.

## Supplementary information

Supplementary Figure Legends

Supplementary Figure 1

Supplementary Figure 2

Supplementary Figure 3

Supplementary Figure 4

Supplementary Figure 5

Supplementary Table 1

Supplementary Table 2

## References

[1] Siegel RL, Miller KD, Jemal A (2020). Cancer statistics, 2020. CA Cancer J Clin.

[2] DeSantis CE, Miller KD, Goding Sauer A, Jemal A, Siegel RL (2019). Cancer statistics for African Americans, 2019. CA Cancer J Clin.

[3] van Bokhoven A, Varella-Garcia M, Korch C, Johannes WU, Smith EE, Miller HL (2003). Molecular characterization of human prostate carcinoma cell lines. Prostate.

[4] Wu X, Gong S, Roy-Burman P, Lee P, Culig Z (2013). Current mouse and cell models in prostate cancer research. Endocr Relat Cancer.

[5] Seim I, Jeffery PL, Thomas PB, Nelson CC, Chopin LK (2017). Whole-genome sequence of the metastatic PC3 and LNCaP human prostate cancer cell lines. G3.

[6] van Deursen JM (2014). The role of senescent cells in ageing. Nature.

[7] Gu Y, Kim KH, Ko D, Srivastava S, Moul JW, McLeod DG (2005). Androgen and androgen receptor antagonist responsive primary African-American benign prostate epithelial cell line. Anticancer Res.

[8] Kogan I, Goldfinger N, Milyavsky M, Cohen M, Shats I, Dobler G (2006). hTERT-immortalized prostate epithelial and stromal-derived cells: an authentic in vitro model for differentiation and carcinogenesis. Cancer Res.

[9] Shao G, Balajee AS, Hei TK, Zhao Y (2008). p16INK4a downregulation is involved in immortalization of primary human prostate epithelial cells induced by telomerase. Mol Carcinog.

[10] Ke XS, Qu Y, Goldfinger N, Rostad K, Hovland R, Akslen LA (2008). Epithelial to mesenchymal transition of a primary prostate cell line with switches of cell adhesion modules but without malignant transformation. PLoS ONE.

[11] Berger R, Febbo PG, Majumder PK, Zhao JJ, Mukherjee S, Signoretti S (2004). Androgen-induced differentiation and tumorigenicity of human prostate epithelial cells. Cancer Res.

[12] Sherr CJ, DePinho RA (2000). Cellular senescence: mitotic clock or culture shock?. Cell.

[13] Wright WE, Shay JW (2001). Cellular senescence as a tumor-protection mechanism: the essential role of counting. Curr Opin Genet Dev.

[14] Graham MK, Principessa L, Antony L, Meeker AK, Isaacs JT (2017). Low p16. Prostate.

[15] Haga K, Ohno S, Yugawa T, Narisawa-Saito M, Fujita M, Sakamoto M (2007). Efficient immortalization of primary human cells by p16INK4a-specific short hairpin RNA or Bmi-1, combined with introduction of hTERT. Cancer Sci.

[16] Bhatia B, Jiang M, Suraneni M, Patrawala L, Badeaux M, Schneider-Broussard R (2008). Critical and distinct roles of p16 and telomerase in regulating the proliferative life span of normal human prostate epithelial progenitor cells. J Biol Chem.

[17] Quelle DE, Zindy F, Ashmun RA, Sherr CJ (1995). Alternative reading frames of the INK4a tumor suppressor gene encode two unrelated proteins capable of inducing cell cycle arrest. Cell.

[18] Sotillo E, Garriga J, Padgaonkar A, Kurimchak A, Cook JG, Graña X (2009). Coordinated activation of the origin licensing factor CDC6 and CDK2 in resting human fibroblasts expressing SV40 small T antigen and cyclin E. J Biol Chem.

[19] Härmä V, Virtanen J, Mäkelä R, Happonen A, Mpindi JP, Knuuttila M (2010). A comprehensive panel of three-dimensional models for studies of prostate cancer growth, invasion and drug responses. PLoS One.

[20] Drost J, Karthaus WR, Gao D, Driehuis E, Sawyers CL, Chen Y (2016). Organoid culture systems for prostate epithelial and cancer tissue. Nat Protoc.

[21] Zhao Z, Kurimchak A, Nikonova AS, Feiser F, Wasserman JS, Fowle H (2019). PPP2R2A prostate cancer haploinsufficiency is associated with worse prognosis and a high vulnerability to B55α/PP2A reconstitution that triggers centrosome destabilization. Oncogenesis.

[22] Shalem O, Sanjana NE, Hartenian E, Shi X, Scott DA, Mikkelson T (2014). Genome-scale CRISPR-Cas9 knockout screening in human cells. Science.

[23] Keskin H, Garriga J, Georlette D, Graña X (2012). Complex effects of flavopiridol on the expression of primary response genes. Cell Div.

[24] Testa JR, Getts LA, Salazar H, Liu Z, Handel LM, Godwin AK (1994). Spontaneous transformation of rat ovarian surface epithelial cells results in well to poorly differentiated tumors with a parallel range of cytogenetic complexity. Cancer Res.

[25] Flejter WL, Li FP, Antman KH, Testa JR (1989). Recurring loss involving chromosomes 1, 3, and 22 in malignant mesothelioma: possible sites of tumor suppressor genes. Genes Chromosomes Cancer.

[26] Pei J, Jhanwar SC, Testa JR (2012). Chromothripsis in a case of TP53-deficient chronic lymphocytic leukemia. Leuk Res Rep..

[27] Lau L, Gray EE, Brunette RL, Stetson DB (2015). DNA tumor virus oncogenes antagonize the cGAS-STING DNA-sensing pathway. Science.

[28] Demidenko ZN, Blagosklonny MV (2004). Flavopiridol induces p53 via initial inhibition of Mdm2 and p21 and, independently of p53, sensitizes apoptosis-reluctant cells to tumor necrosis factor. Cancer Res.

[29] Boehm JS, Hession MT, Bulmer SE, Hahn WC (2005). Transformation of human and murine fibroblasts without viral oncoproteins. Mol Cell Biol.

[30] Dannenberg JH, van Rossum A, Schuijff L, te Riele H (2000). Ablation of the retinoblastoma gene family deregulates G(1) control causing immortalization and increased cell turnover under growth-restricting conditions. Genes Dev.

[31] Badal S, Aiken W, Morrison B, Valentine H, Bryan S, Gachii A (2020). Disparities in prostate cancer incidence and mortality rates: solvable or not?. Prostate.

[32] Badal S, Campbell KS, Valentine H, Ragin C (2019). The need for cell lines from diverse ethnic backgrounds for prostate cancer research. Nat Rev Urol.

[33] Beauséjour CM, Krtolica A, Galimi F, Narita M, Lowe SW, Yaswen P (2003). Reversal of human cellular senescence: roles of the p53 and p16 pathways. EMBO J.

[34] Wei W, Hemmer RM, Sedivy JM (2001). Role of p14(ARF) in replicative and induced senescence of human fibroblasts. Mol Cell Biol.

[35] Robinson D, Van Allen EM, Wu YM, Schultz N, Lonigro RJ, Mosquera JM (2015). Integrative clinical genomics of advanced prostate cancer. Cell.

[36] Network CGAR. (2015). The molecular taxonomy of primary prostate cancer. Cell.

[37] Gao J, Aksoy BA, Dogrusoz U, Dresdner G, Gross B, Sumer SO (2013). Integrative analysis of complex cancer genomics and clinical profiles using the cBioPortal. Sci Signal.

[38] Cerami E, Gao J, Dogrusoz U, Gross BE, Sumer SO, Aksoy BA (2012). The cBio cancer genomics portal: an open platform for exploring multidimensional cancer genomics data. Cancer Disco.

