# Supplementary material for: Immortalization of human primary prostate epithelial cells via CRISPR inactivation of the CDKN2A locus and expression of telomerase
Source: Prostate Cancer Prostatic Dis. 2020 Sep 1;24(1):233–43. doi: 10.1038/s41391-020-00274-4 (PMC7917161; doi:10.1038/s41391-020-00274-4)
Supplement: Supplementary file 1 — Supplementary Figure Legends [file 41391_2020_274_MOESM1_ESM.docx]

**Supplementary Figures**

**Suppl. Fig. 1. T-ΔN2A clones 1 and 2 do not express AR, and express the p63 basal cell marker. A** T-ΔN2A clones 1 and 2 grown on coverslips dot not express AR. LNCaP and PC3 PCa cells were used as positive and negative controls, respectively. **B**, Additional characterization of T-ΔN2A clones 1 and 2 showing pRB and p63 status.

**Suppl. Fig. 2. CMA of T-ΔN2A Clone 1.** CMA was performed as described in materials and methods. Blue arrows indicate gain and loses presumably occurring during clone selection. Green arrows indicate likely polymorphisms, as they are detected in both clones. The CDKN2A deletion is indicated by an orange arrow. r[hg19]: 5q23.2q35.3(121,598,528-180,719,789)x3, 9p21.3(21,976,765-22,005,191)x1, 19p13.3(1-683,931)x1.

**Suppl. Fig. 3. CMA of T-ΔN2A Clone 2.** CMA was performed as described in materials and methods. Blue arrows indicate gain and loses presumably occurring during clone selection. Green arrows indicate likely polymorphisms, as they are detected in both clones. The CDKN2A deletion is indicated by an orange arrow. arr[hg19]: (2p)x3, 4q34.3q35.2(181,652,075-190,957,473)x1, 9p21.3(21,976,285-22,006,225)x0, 9q31.1q34.3(105,593,380-141,020,389)x3, (20q)x3.

**Suppl. Fig 4.** Fluorescence in situ hybridization (FISH) analysis of multiple subclones from Clone 1 of human prostate epithelial cells (hPrEC) that originally exhibited a mosaic karyotype, 46,XY,der(19)t(5;19)(q23.2;p13.3)[7]/46,XY[4]. Since Clone 1 had a significant proportion of karyotypically normal cells, a chromosome 5q32 *PDGFRB* break apart FISH probe was used to screen 11 subclones of Clone 1 to identify a karyotypically normal subclone. **A**, Gallery of 18 nuclei from representative subclone (#11), each showing 3 fused orange-green signals, indicating three copies of the *PDGFRB* locus at 5q32. **B**, Three representative nuclei from each of 10 of the 11 subclones screened by FISH, along with a summary showing a high percentage of nuclei with three copies of *PDGFRB* in each subclone (200 nuclei scored per subclone). Note: to more readily count FISH signals, DAPI staining is not superimposed here, and instead a blue line encircles the area encompassed by each nucleus.

**Suppl. Fig. 5. The immortalized human prostate epithelial cell lines have intact p53 pathway and normal contact inhibition.** Upregulation of p53 in immortalized hPrEC-*T-ΔN2A* clones upon flavopiridol treatment.
