# Supplementary figures and images for: Immortalization of human primary prostate epithelial cells via CRISPR inactivation of the CDKN2A locus and expression of telomerase
Source: Prostate Cancer Prostatic Dis. 2020 Sep 1;24(1):233–43. doi: 10.1038/s41391-020-00274-4 (PMC7917161; doi:10.1038/s41391-020-00274-4)

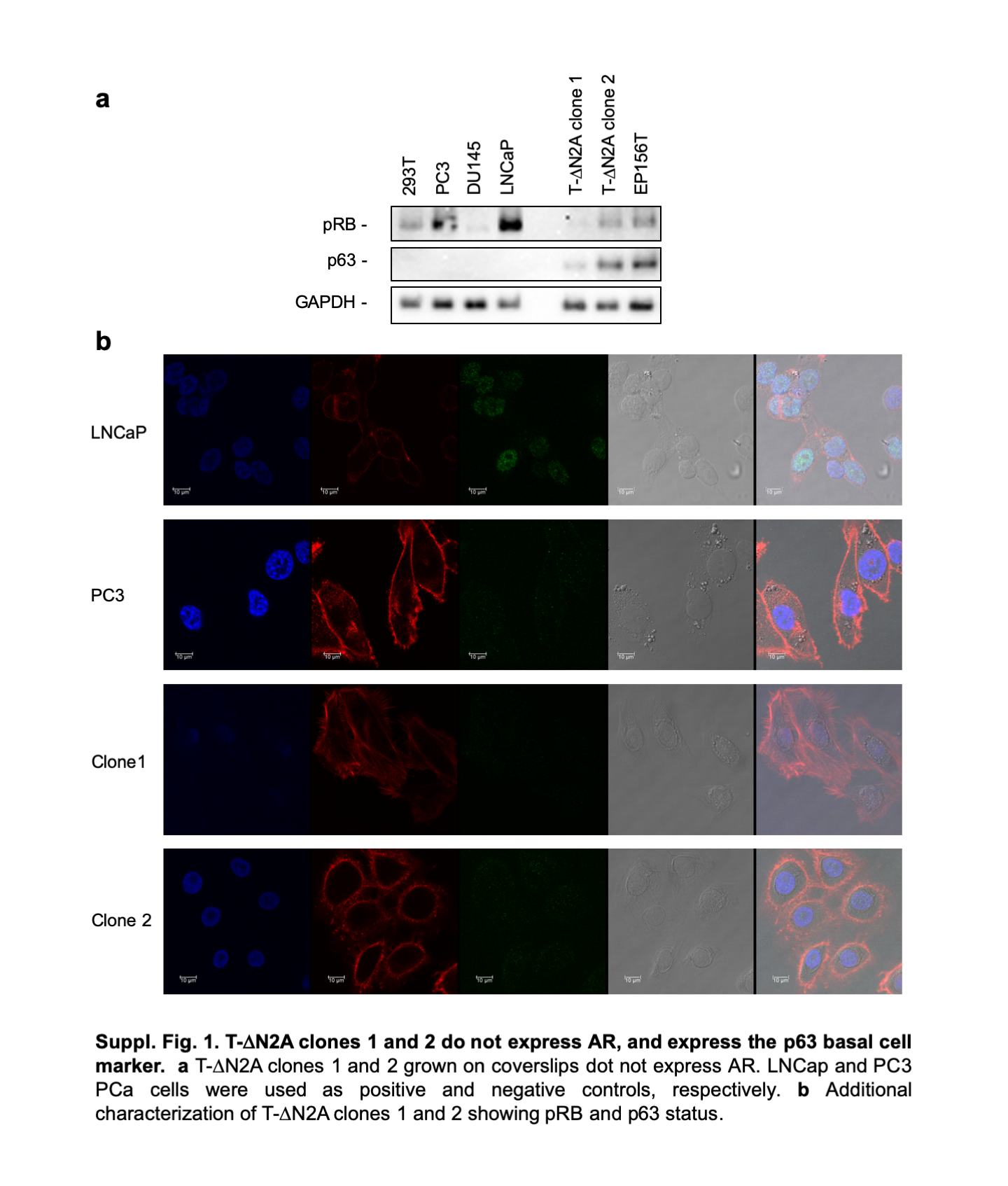

Supplement: Supplementary file 2 — Supplementary Figure 1 [file 41391_2020_274_MOESM2_ESM.tif]

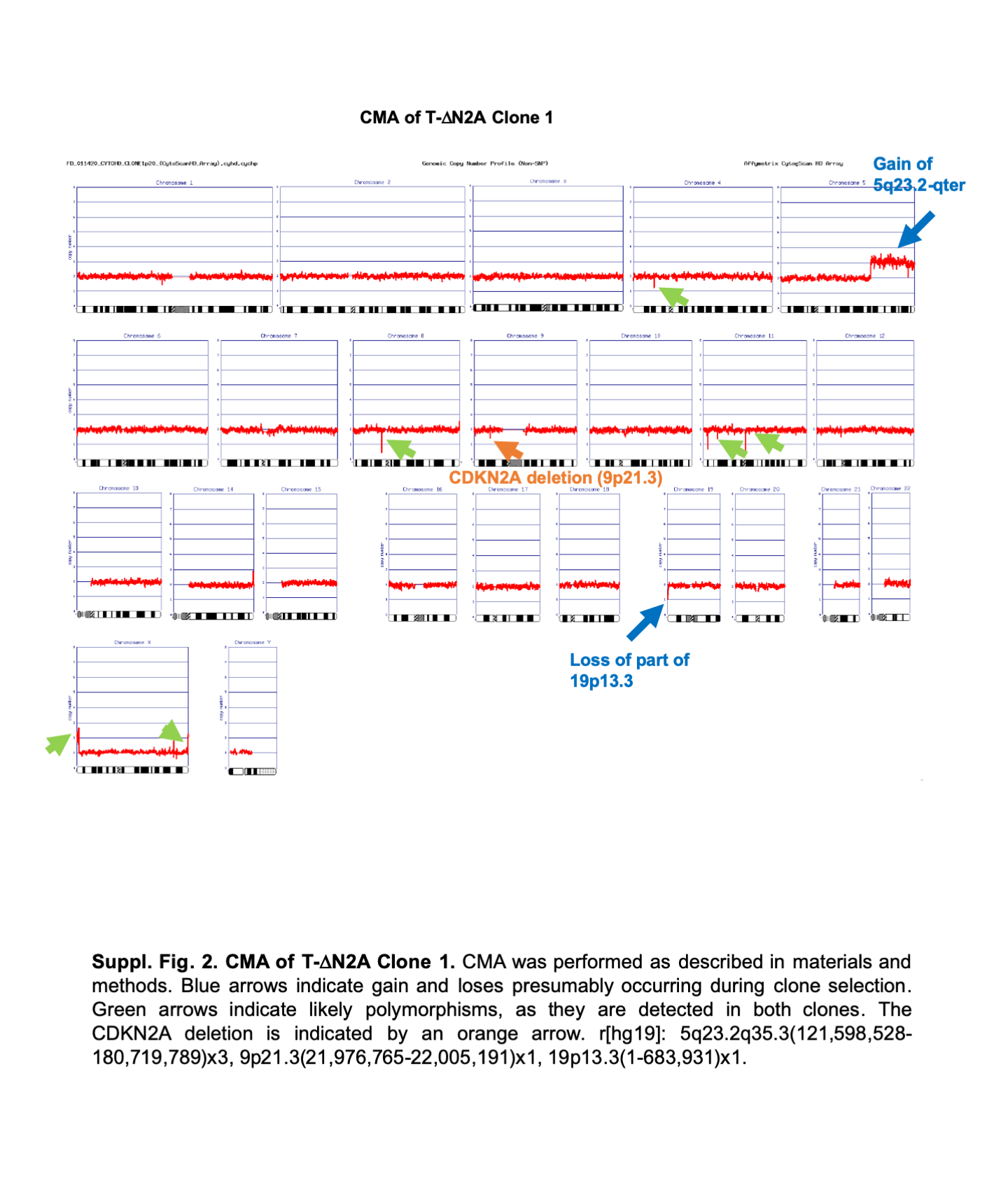

Supplement: Supplementary file 3 — Supplementary Figure 2 [file 41391_2020_274_MOESM3_ESM.tif]

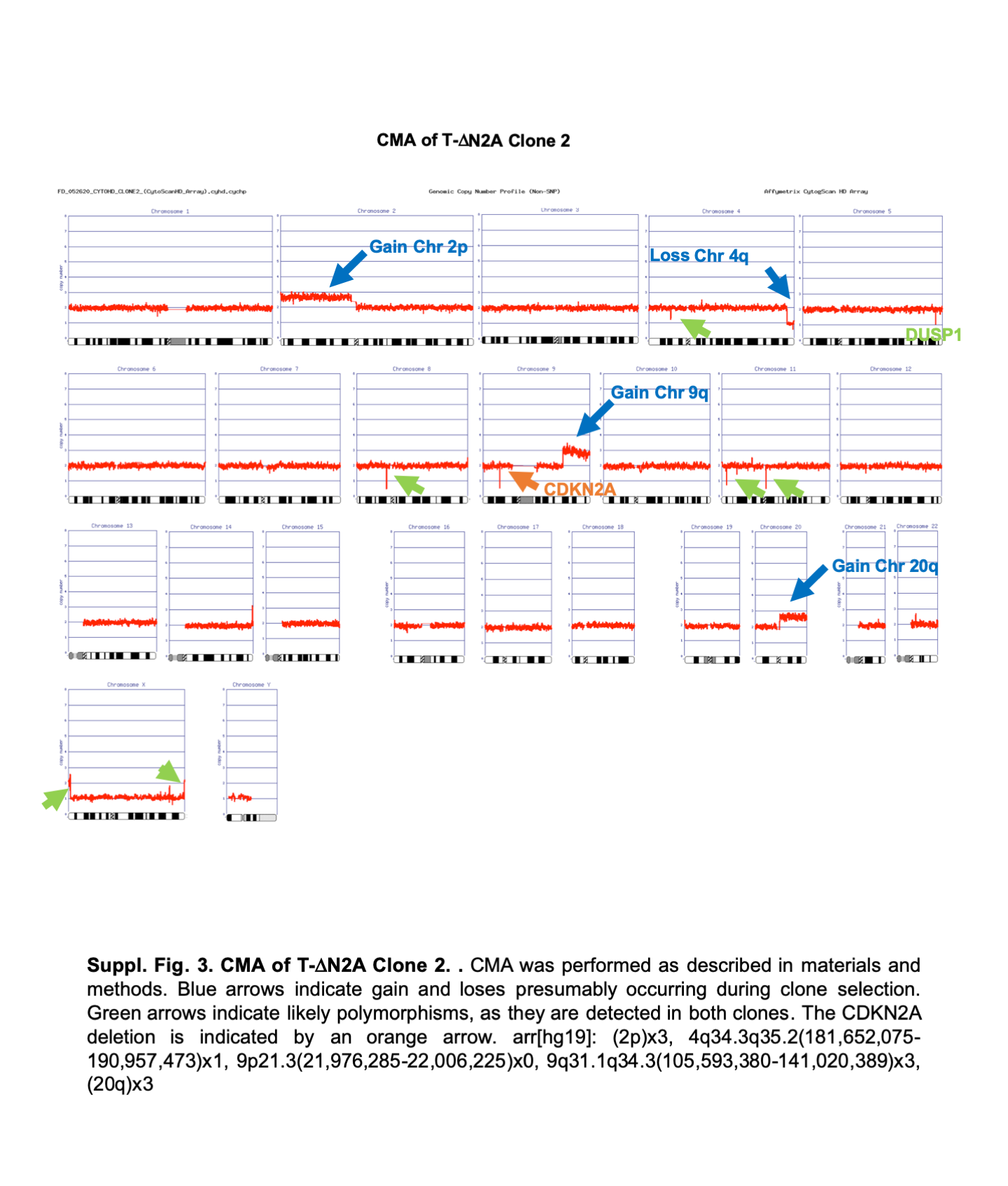

Supplement: Supplementary file 4 — Supplementary Figure 3 [file 41391_2020_274_MOESM4_ESM.tif]

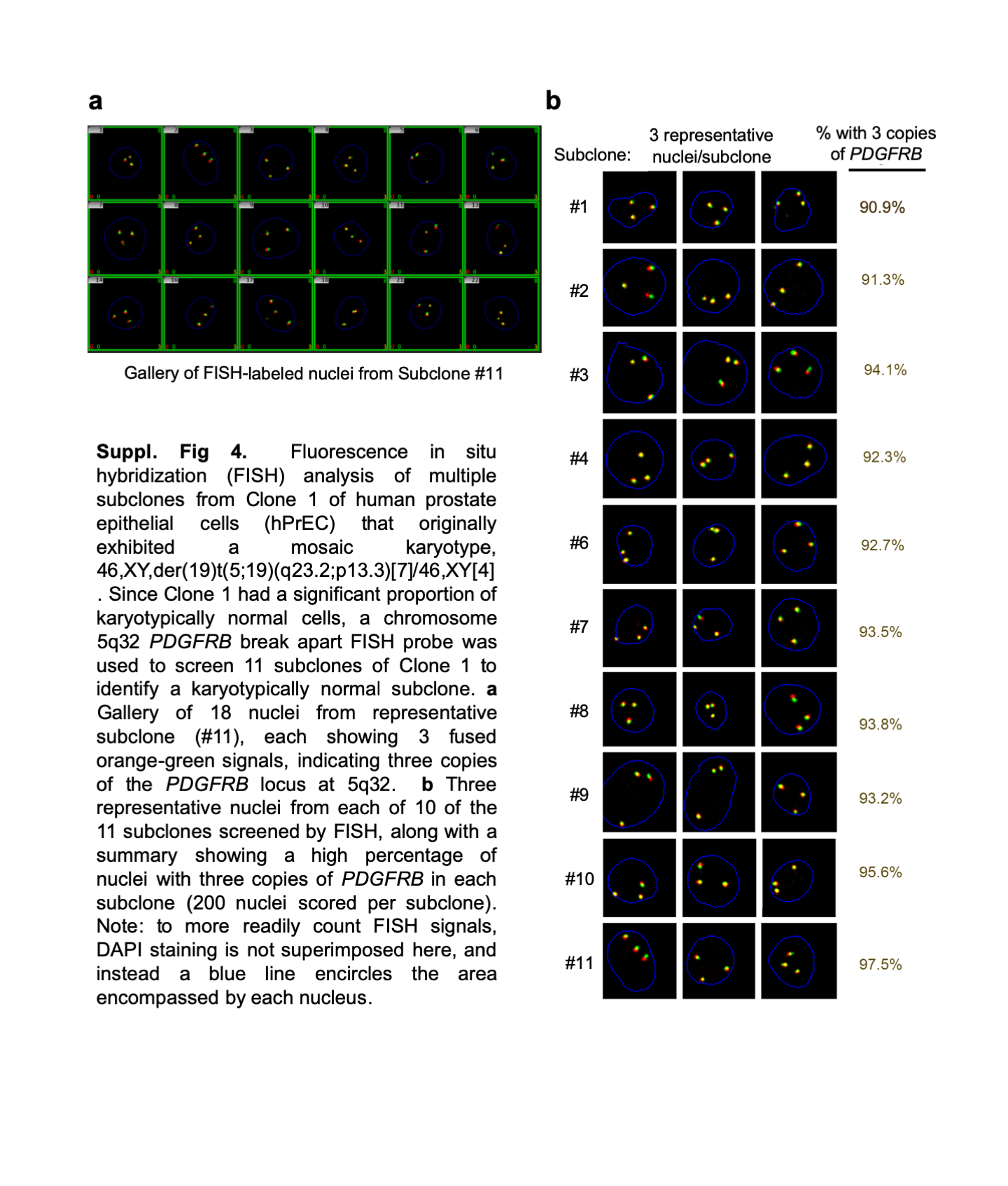

Supplement: Supplementary file 5 — Supplementary Figure 4 [file 41391_2020_274_MOESM5_ESM.tif]

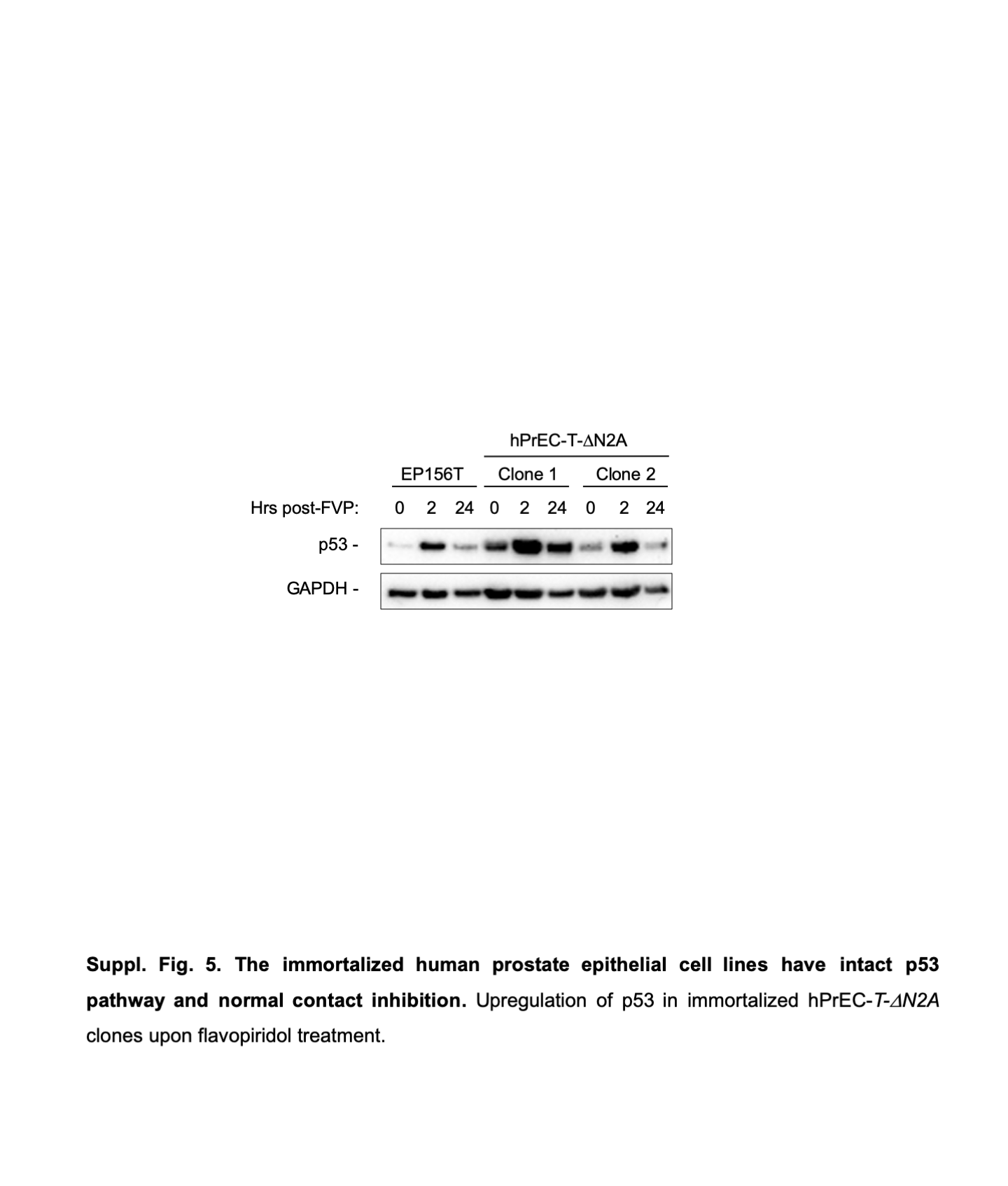

Supplement: Supplementary file 6 — Supplementary Figure 5 [file 41391_2020_274_MOESM6_ESM.tif]
